# Garlic-derived compound S-allylmercaptocysteine inhibits cell growth and induces apoptosis via the JNK and p38 pathways in human colorectal carcinoma cells

**DOI:** 10.3892/ol.2014.2579

**Published:** 2014-09-30

**Authors:** YAN ZHANG, HONG-YAN LI, ZHI-HUA ZHANG, HONG-LEI BIAN, GUI LIN

**Affiliations:** 1 Department of Colorectal Surgery, Third Hospital of Hebei Medical University, Shijiazhuang, Hebei 050051, P.R. China; 2 Department of English, Hebei University of Science and Technology, Shijiazhuang, Hebei 050051, P.R. China

**Keywords:** human colorectal carcinoma cells, SW620, p53, S-allylmercaptocysteine, c-Jun N-terminal kinase, signal pathway, p38

## Abstract

S-allylmercaptocysteine (SAMC) is an active compound that is derived from garlic and has been demonstrated to possess antitumor properties *in vitro*. The present study aimed to investigate the effect of SAMC and determine the underlying mechanism of this effect on human colorectal carcinoma cells. The SW620 cells were cultured with various concentrations of SAMC and cell viability was detected using an MTT assay. Analysis of apoptosis was performed using terminal deoxynucleotidyl-transferase-mediated deoxyuridine triphosphate nick end labeling. The c-Jun N-terminal kinase (JNK) and p38 mitogen activated protein kinase (p38) signaling pathways were investigated by polymerase chain reaction. SAMC was observed to reduce cell viability in a dose- and time-dependent manner, partially through the induction of apoptosis in human colorectal carcinoma cells. At the molecular level, SAMC induces apoptosis through JNK and p38 signaling pathways, increasing tumor protein p53 (p53) and Bax activation in the SW620 cells. The most effective concentration of SAMC for the induction of SW620 cell apoptosis was found to be 400 μM, which was confirmed through cell viability assays and apoptosis analysis. The current study indicated that SAMC inhibits cell proliferation and induces apoptosis of SW620 cells via the JNK and p38 pathways. The results from the current study demonstrated that SAMC must be further investigated as a novel preventive or therapeutic agent for the treatment of colorectal carcinoma, and potentially for use in other tumor types.

## Introduction

Garlic (*Allium sativum*) is a vegetable of the Allium class of bulb-shaped plants, which has been used for thousands of years and in numerous cultures as a food and for its medicinal purposes, dating back >4,000 years (1). Currently, garlic is used to aid in the prevention of heart disease, high cholesterol and blood pressure and to boost the immune system (2–4). However, evidence from epidemiological and experimental carcinogenesis studies has indicated that certain components of garlic possess anticancer activity (5). Subsequent to reacting with endogenous antioxidants, including cysteine and reduced glutathione, the majority of garlic allyl sulfides, which are absorbed in the gastrointestinal tract, have also been reported to biotransform to the corresponding allylmercapto glutathione S-congugate (6). One of these allylmercapto glutathione S-conjugates, S-allylmercapto-L-cysteine (SAMC), is a water soluble organosulfur compound that is found in aged garlic extract and is obtained through the ethanol extraction of sliced garlic bulbs. SAMC has been reported to exert an inhibitory effect on tumorigenesis (7), however, the mechanisms through which this occurs are poorly understood. The current study investigates the effects of SAMC on the growth of the SW620 human colorectal carcinoma cell line.

## Materials and methods

### Cell culture and maintenance

The human colorectal carcinoma SW620 cell line was obtained from the Peking Union Medical College (Beijing, China), and maintained in minimal RPMI 1640 medium (Life Technologies, Carlsbad, CA, USA) containing 10% fetal bovine serum (FBS; Life Technologies) and 1% antibioticantimycotic (Life Technologies) in a humidified atmosphere of 5% CO_2_ at 37°C.

### Drug treatment

SAMC (06-284) was provided by Wakunaga Pharmaceutical Co., Ltd. (Osaka, Japan). According to the manufacturer’s instructions, sterilized stock solutions of SAMC (5 mM) were freshly prepared in phosphate-buffered saline (PBS), and refrigerated at 4°C. The SW620 cells were seeded at a density of 1×10^4^ cells/well in 24-well plates and incubated for 24 h. SAMC was dissolved in PBS and added to the culture media at various concentrations, ranging from 0–450 μM, and the cells were subsequently incubated for 72 h.

### Cell viability assay

The SW620 cells were plated in 96-well plates at a density of 1×10^4^ cells/well. The cells were incubated with various concentrations of SAMC (0, 100, 200, 300, 350, 400 and 450 μM) for 12, 24, 48 and 72 h. MTT solution [0.5 g 3-(4,5-dimethylthiazol-2-yl)-2,5-diphenyltetrazolium bromide in 100 ml PBS; Sigma, St. Louis, MO, USA] was added to the culture medium (final concentration, 500 μg/ml) 4 h prior to the completion of the treatment and the reaction was terminated by the addition of 100 μl of 10% acidified sodium dodecyl sulfate to the cell culture. The absorbance value (A) was measured at 570 nm using a multiwell spectrophotometer (Bio-Rad 550; Bio-Rad, Hercules, CA, USA). The percentage of cell inhibition was calculated using the following formula: Inhibitory rate (%) = (1 − A of experiment well/A of control well) × 100. A dose-survival curve was obtained for each experiment. The experiments were performed in triplicate.

### Apoptosis analysis

Apoptosis of SW620 cells was detected using terminal deoxynucleotidyl transferase-mediated deoxyribonucleotide triphosphate-digoxigenin nick-end labeling (TUNEL), following drug treatment for 72 h. Briefly, the cells were fixed for 1 h at room temperature and subsequently incubated in permeabilization solution for 2 min on ice. The subsequent staining was performed according to the manufacturer’s instructions.

### Quantitative polymerase chain reaction (PCR)

The expression of components of the c-Jun N-terminal kinase (JNK) and p38 mitogen activated protein kinase (p38) pathways was assessed using reverse transcription (RT)-PCR and quantitative-PCR. Briefly, following treatment with SAMC, the cells were washed with cold PBS and the total RNA from the cells was isolated using TRIzol reagent (Life Technologies). The RNA was then reverse transcribed into the complementary (c)DNA for PCR amplification. The primers used were designed using the software Primer Premier version 5.0 (Premier Biosoft, Palo Alto, CA, USA) and synthesized by SBS Genetech Co., Ltd. (Beijing, China). Further information with regard to the gene-specific primer pairs used is listed in Table I. The PCR products were detected by 2.5% agarose gel electrophoresis. Quantitative RT-PCR analysis was performed according to the protocol detailed in a previous study (8). Briefly, 500 ng of the total RNA was reverse-transcribed to cDNA using Takara PCR Thermal Cycler Dice (Takara Biotechnology (Dalian) Co., Ltd., Dalian, China) and subsequently the cDNA was subjected to quantitative-PCR using the Applied Biosystems 7500 PCR device (Applied Biosystems, Foster City, CA, USA). mRNA expression of the components of the JNK and p38 pathways was normalized using GAPDH mRNA and the data were analyzed according to relative gene expression using the 2^−ΔΔCT^ (Livak) method. The experiments were performed in triplicate.

### Statistical analysis

Statistical analyses of the data were performed using a one-way analysis of variance followed by the Tukey-Kramer honest significant difference (HSD) test for the three sets of results. P<0.05 was considered to indicate a statistically significant difference. Statistical analyses were performed using JMP^®^ Statistical Discovery Software version 4.0 (SAS Institute, Cary, NC).

## Results

### SAMC-induced reduction of SW620 cell viability

**Figure 1:**
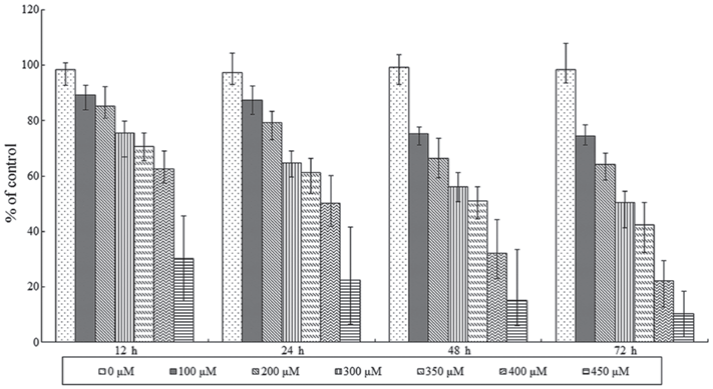
SAMC in reduces viability of human colorectal carcinoma cells. The cells were grown and treated with various concentrations of SAMC for up to 72 h. Cell viability was analyzed using the MTT assay. The data are presented as the mean ± standard error (n=6) of two independent experiments performed in triplicate. Control vs. treated cells, ^*^P<0.05 and ^**^P<0.01. SAMC, S-allylmercaptocysteine.

Viability of SAMC-treated SW620 cells was initially assessed using an MTT assay. The results revealed that SAMC decreased tumor cell viability in a dose- and time-dependent manner (Fig. 1). However, cell death was observed when the concentration of SAMC was >450 μM.

### Apoptosis analysis

**Figure 2:**
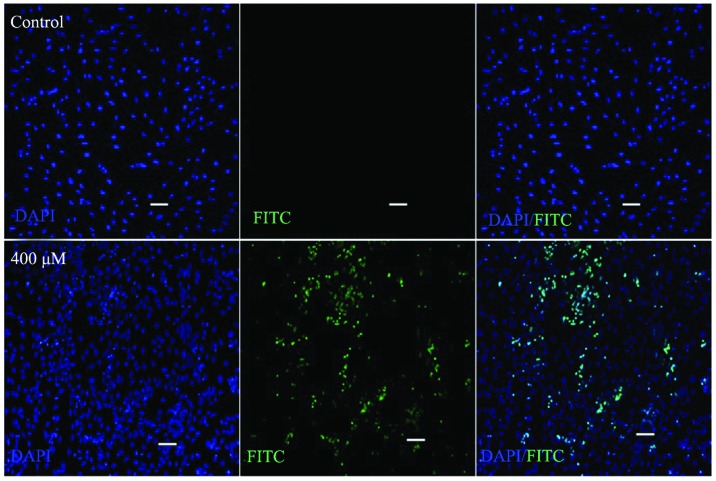
Effect of 400 μM of SAMC on apoptosis of human colorectal carcinoma SW620 cells after 48 h, using the TUNEL method. TUNEL is a common method for detecting DNA fragmentation that results from apoptotic signaling cascades. Positive cells were stained green, indicating that DNA fragmentation had taken place. This symbolic apoptotic event was greatly reduced in the presence of SAMC (Bar 50 μm). SAMC, S-allylmercaptocysteine; TUNEL, terminal deoxynucleotidyl-transferase-mediated deoxyuridine triphosphate nick end labelling; DAPI, 4′,6-diamidino-2-phenylindole; FITC, fluorescein isothiocyanate.

SW620 cells were treated with SAMC at various concentrations, and subsequently cultured for 72 h. TUNEL is a common method for detecting DNA fragmentation that results from apoptotic signaling cascades. DNA fragmentation was labeled *in situ* via a TUNEL assay. A significant increase in the proportion of DNA fragmentation-positive cells was observed following SAMC treatment, in contrast with the control group (Fig. 2).

### JNK and p38 pathway assay through PCR

**Figure 3:**
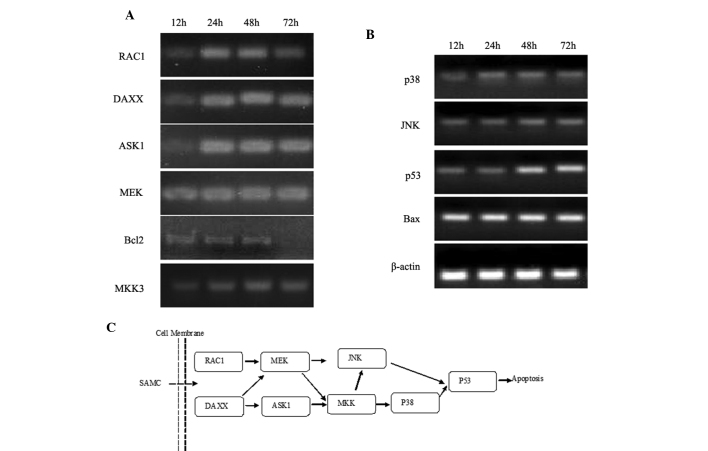
Reverse transcriptase-polymerase chain reaction analysis of the expression of JNK and p38 pathway members. (A and B) Expression of the members of the JNK and p38 pathways in SW620 cells 72 h following treatment with SAMC (400 μM). (C) The process of the regulation of cell apoptosis by the JNK and p38 signal pathways. JNK, c-Jun N-terminal kinase; SAMC, S-allylmercaptocysteine; RAC1, Ras-related C3 botulinum toxin substrate 1; DAXX, death-associated protein 6; MEK, mitogen-activated protein kinase kinase; ASK1, Apoptosis signal-regulating kinase 1; MKK3, mitogen-activated protein kinase kinase 3; p38, p38 mitogen activated protein kinase; p53, tumor protein p53.

**Figure 4:**
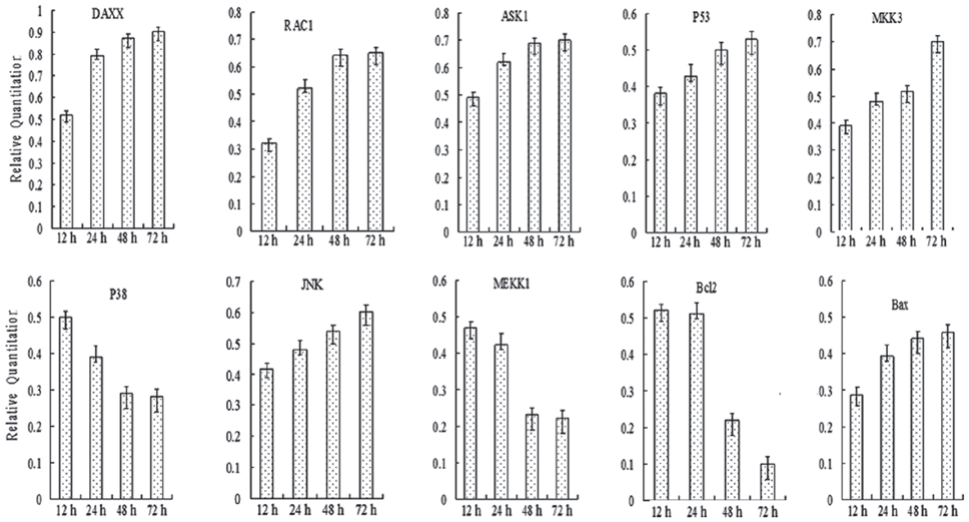
Quantitative polymerase chain reaction analyses of the expression of JNK and p38 pathway members in SW620 cells at 72 h following treatment with SAMC (400 μM). Expression of RAC1, DAXX, MEKK1, ASK1, JNK, MKK3, p38, p53, Bcl2 and Bax was detected, and the gene expression level exhibited a time-lapse increase. However, the gene expression of Bcl2 exhibited a time-lapse decrease. JNK, c-Jun N-terminal kinase; SAMC, S-allylmercaptocysteine; RAC1, Ras-related C3 botulinum toxin substrate 1; DAXX, death-associated protein 6; MEKK1, mitogen-activated protein kinase kinase 1; ASK1, Apoptosis signal-regulating kinase 1; MKK3, mitogen-activated protein kinase kinase 3; p38, p38 MAP kinase; p53, tumor protein p53, Bcl2, B-cell lymphoma 2; Bax, Bcl2-like protein 4.

To investigate the roles of the JNK and p38 pathways in SAMC-induced apoptosis, the mRNA levels of the members of the JNK and p38 pathways in SAMC treated SW620 cells were determined. Cellular total RNA was isolated following 24, 48 and 72 h of treatment with SAMC respectively, and RT-PCR and quantitative PCR were performed for members of JNK and p38 pathway, including RAC1, DAXX, MEKK1, ASK1, JNK, MKK3, p38, p53, Bcl-2 and Bax. The PCR assay demonstrated that following incubation with SAMC, the specific genes of the JNK and p38 apoptosis pathway, including RAC1, DAXX, MEKK1, ASK1, JNK, MKK3, p38, p53, Bcl-2 and Bax could be detected. Predominantly, the gene expression leved showed a time-lapse increase, however, the gene expression of Bcl-2 showed a time-lapse decrease (Figs. 3 and 4).

## Discussion

Garlic has been shown to be effective against a broad spectrum of diseases and SAMC, one of the constituents of garlic, has been proposed to be responsible for this biological activity (9). In the current study, the results clearly demonstrated that SAMC induces apoptosis in the human colorectal carcinoma cell line SW620 *in vitro*, which may explain the antiproliferative activity of garlic. These results also suggest that the induction of apoptotic cell death by SAMC occurs via the JNK and p38 pathways that activate p53 and Bax. Apoptosis is a systematically regulated process that involves the expression of numerous gene products. Of the major genes that regulate apoptosis, the antiapoptotic Bcl-2 gene and the proapoptotic Bax gene are of particular interest. p53 is a tumor suppressor gene and a sequence-specific transcription factor; p53 activation may occur through a variety of forms of cellular stress (10). Tumor-derived p53 mutants that were able to promote cell growth and transformation were the focus of the majority of initial studies of p53 (11,12) and subsequently, p53 was determined as an essential mediator of cell cycle arrest in response to various cellular stresses (13). Experiments that introduced p53 into a p53-deficient leukemia cell line >10 years after its discovery provided the first evidence that p53 could promote apoptosis (14). The results obtained by Basu and Haldar indicated that a loss of p53 function may substitute for elevated Bcl-2 activity in breast cancer cells as well as suggesting that p53 may be able to downregulate Bcl-2 (15). As it is often highly dependent on cell context, a common theme of cell regulation is crosstalk between various signaling pathways. Numerous stimuli simultaneously activate the JNK and p38 pathways as several upstream regulators are shared between the two pathways (16). Increased activation of p38 inhibition by JNK has also been observed in mouse models. For example, increased activation of the JNK pathway accounts for p38-deficient myoblasts not exiting the cell cycle or proliferating in differentiation-promoting conditions (17). JNK activation has been identified in samples from human gastric cancer. Similarly, in a mouse model of gastric cancer caused by methyl-nitroso-urea treatment, JNK has been found to control tumor initiation and promotion by affecting cell proliferation and the production of reactive oxygen species (ROS) (18). The induction of apoptosis by numerous types of cellular stress also involves p38. These effects can be mediated by transcriptional and post-transcriptional mechanisms, which affect death receptors, survival pathways or pro- and antiapoptotic Bcl-2 proteins. The contribution of these various mechanisms to p38-induced apoptosis is likely to be regulated in a stimulus- and context-dependent manner (19). p38 activation is occasionally triggered by apoptotic stimuli through secondary routes, including the production of ROS. It is likely that this mechanism is significant in the suppression of p38-mediated tumor initiation, which triggers apoptosis in response to the expression of oncogenes that induce ROS in immortalized cells (20). The results presented in the current study provide a mechanistic basis for the antiproliferative effects of SAMC and partially elucidate the chemopreventive action of SAMC that has been reported in previous studies (21,22). The present results also provide a mechanistic basis for the previously observed effects of SAMC on human colorectal carcinoma cells as the JNK and p38 pathways were revealed to regulate the apoptosis of cells through the activation of the p53 pathway.

In conclusion, the present study provides evidence that the garlic derivative SAMC inhibits the growth of cancer cells *in vitro* by directly activating the p53 pathway. The current study also provided evidence that SAMC-induced apoptosis is related, at least in part, to the activation of the JNK and p38 pathways; however, additional signaling mechanisms have yet to be elucidated. These results indicate that SAMC or related compounds may provide a novel approach to cancer chemoprevention and therapy, and may encourage the development of more potent derivatives of SAMC.

## Figures and Tables

**Table I:** Primer sequences used in reverse transcription-PCR and quantitative PCR assays.

Gene	Direction	Primer sequence	Tm, °C	Cycle	Fragment size, bp
RAC1	F	5′ AAACCGGTGAATCTGGGCTT 3′	60.0	30	91
	R	5′ AGAACACATCTGTTTGCGGA 3′			
DAXX	F	5′ GCTTAGTTGCATGAAGGCGG 3′	60.0	30	149
	R	5′ AGAATTCCTGCTCAGAAACCGT 3′			
ASK1	F	5′ CATGTCAACCGGGATGTCCA 3′	58.0	30	169
	R	5′ CTAGACCCGTACTGCTGCTG 3′			
MEKK1	F	5′ AAGCCTGCCGGTGACTAAC 3′	60.0	30	116
	R	5′ GCATCACCCGGAGGAGAAAT 3′			
MKK3	F	5′ GAAAGCCTGCCGGTGACTAA 3′	60.0	30	200
	R	5′ TTCCCGTTCTCAGCCTTGAC 3′			
JNK	F	5′ CTGAAGCAGAAGCTCCACCA 3′	60.0	30	159
	R	5′ CACCTAAAGGAGAGGGCTGC 3′			
p38	F	5′ ATGAAGCTCTCCAACACCCG 3′	60.0	30	205
	R	5′ GCACCTAAAGGAGAGGGCTG 3′			
p53	F	5′ CAGCCCTCTCCTTTAGGTGC 3′	57.5	30	137
	R	5′ GCTGCTGCTTCTAGACTGCT 3′			
Bcl2	F	5′ GCTCTCCAACACCCGTACAT 3′	60.0	30	203
	R	5′ GCTGCACCTAAAGGAGAGGG 3′			
Bax	F	5′ AGGGTGTAAAACGCAGCTCA 3′	58.7	30	202
	R	5′ AGGGTGTAAAACGCAGCTCA 3′			
β-actin	F	5′ AATGGGCAGCCGTTAGGAAA 3′	60.0	30	169
	R	5′ GCGCCCAATACGACCAAATC 3′			

PCR, polymerase chain reaction; F, forward; R, reverse.
